# Dendritic Cells in Chronic Mycobacterial Granulomas Restrict Local Anti-Bacterial T Cell Response in a Murine Model

**DOI:** 10.1371/journal.pone.0011453

**Published:** 2010-07-06

**Authors:** Heidi A. Schreiber, Paul D. Hulseberg, JangEun Lee, Jozsef Prechl, Peter Barta, Nora Szlavik, Jeffrey S. Harding, Zsuzsanna Fabry, Matyas Sandor

**Affiliations:** 1 Department of Pathology and Laboratory Medicine, University of Wisconsin, School of Medicine and Public Health, Madison, Wisconsin, United States of America; 2 Cellular and Molecular Pathology Training Program, University of Wisconsin, Madison, Wisconsin, United States of America; 3 Department of Pulmonology, Semmelweis University, Budapest, Hungary; 4 Sejtdiagnosztika Kft, Hospital Bajcsy Zsilinszky, Budapest, Hungary; National Institute for Infectious Diseases L. Spallanzani, Italy

## Abstract

**Background:**

Mycobacterium-induced granulomas are the interface between bacteria and host immune response. During acute infection dendritic cells (DCs) are critical for mycobacterial dissemination and activation of protective T cells. However, their role during chronic infection in the granuloma is poorly understood.

**Methodology/Principal Findings:**

We report that an inflammatory subset of murine DCs are present in granulomas induced by *Mycobacteria bovis* strain Bacillus Calmette-guerin (BCG), and both their location in granulomas and costimulatory molecule expression changes throughout infection. By flow cytometric analysis, we found that CD11c^+^ cells in chronic granulomas had lower expression of MHCII and co-stimulatory molecules CD40, CD80 and CD86, and higher expression of inhibitory molecules PD-L1 and PD-L2 compared to CD11c^+^ cells from acute granulomas. As a consequence of their phenotype, CD11c^+^ cells from chronic lesions were unable to support the reactivation of newly-recruited, antigen 85B-specific CD4^+^IFNγ^+^ T cells or induce an IFNγ response from naïve T cells *in vivo* and *ex vivo*. The mechanism of this inhibition involves the PD-1:PD-L signaling pathway, as *ex vivo* blockade of PD-L1 and PD-L2 restored the ability of isolated CD11c^+^ cells from chronic lesions to stimulate a protective IFNγ T cell response.

**Conclusions/Significance:**

Our data suggest that DCs in chronic lesions may facilitate latent infection by down-regulating protective T cell responses, ultimately acting as a shield that promotes mycobacterium survival. This DC shield may explain why mycobacteria are adapted for long-term survival in granulomatous lesions.

## Introduction

The formation of a granuloma in response to a pathogen creates an immunological foci that contains the antigen. During infection with mycobacteria, the granuloma contains the bacteria, prevents dissemination, and localizes immune responses to limit tissue damage. Though it protects the host, the granuloma also facilitates bacterial survival, which may eventually allow for disease tansmission. Acute mycobacteria-induced granulomas formed early in infection are large lesions with high bacterial burden [1]. They are characterized by the presence of a high proportion of IFNγ-producing CD4^+^ T cells, which are critical activators of microbicidal pathways in bacteria-containing macrophages [2]. Chronic granulomas are smaller, more structured lesions with a lower bacterial load and reduced killing–they contain bacteria and prevent dissemination, but are ultimately unable to sterilize the lesion. In this way the chronic granuloma provides a home for bacterial latency, in which reactivation can occur decades later after immune stress from AIDS, old age, or anti-TNFα therapy, for instance. Reactivated bacteria grow, disseminate and often result in fatality [3], [4]. Mutant mycobacteria strains that induce poor granuloma formation result in increased bacterial growth [5]–[9]. Virulent mycobacteria species have actually evolved gene-specific strategies to promote early granuloma formation and ensure their own *long-term* survival so as to increase chances of disease transmission. While the function of acute granulomas is the focus of intense research, much less is known about chronic granulomas.

DCs are critical in the initiation of immune responses since they are the only antigen presenting cell capable of activating naïve T cells and efficiently initiating a recall T cell response [10]. Following mycobacterial infection, DCs are required for initiation of the adaptive immune response by facilitating dissemination of mycobacteria and mycobacterial antigen from the site of infection to the draining lymph node [11]–[15]. However, little is known about the role of DCs during chronic infection. Immunohistochemistry of chronic granulomas from lungs of tuberculosis patients shows DCs in and around the granuloma [16](*unpublished data*). To track and analyze DCs in sarcoid granulomas, the likely sites for long-term mycobacterial persistence, we used a systemic *Mycobacterium bovis* Bacillius Calmette-guerin (BCG) infection model. This model offers several advantages in the study of latent mycobacterial infections. Lesions from BCG infection are the best characterized of any mycobacterial-induced granulomas models, are technically convenient and numerous to isolate, and very recently have been studied with new and intriguing *in vivo* imaging [17]. Most of the two billion people infected with *Mycobacterium* species control infection by maintaining symptom-free latency of the bacilli in chronic granulomas. Murine infection with *Mycobacterium tuberculosis* results in a sustained and eventually fatal bacterial burden, which does not reflect the low bacterial burden found in chronically infected humans [18], [19]. Murine infection with BCG, however does achieve the low bacterial load observed during human Mtb infection. Furthermore, three billion people have been vaccinated with live BCG, which has been proposed to survive within granulomas, with 100 million people newly vaccinated each year, yet its efficacy remains limited [20]–[22]. The ineffectiveness of the most widely distributed vaccine worldwide certainly warrants rigorous investigation.

In addition, *Mycobacterium bovis* also presents serious health threats of its own. An estimated 1–2% of human tuberculosis cases are caused by *Mycobacterium bovis* in developed countires, while in still developing countries it is 10%, totaling 20–200 million cases worldwide [23]–[25]. With increased immigration into the U.S. from Mexico, this presents a more imminent threat to the U.S., especially in binational border communities [26]. Primarily acquired zoonotically by the consumption of raw meat and unpasteurized milk from infected bovine, there have also been cases of *Mycobacterium bovis* transmission from person to person [25], [27], [28]. *M. bovis* tuberculosis patients are over twice as likely to die during treatment compared to those with *Mycobacterium tuberculosis*, which may be attributed to its universal resistance to the first line tuberculosis drug pyrazinamide [26].

Here, we report data that suggests DCs are critical in the transition from the acute to chronic granulomas. CD11c^+^ cells from acute and chronic granulomas differ in co-stimulatory phenotype, location within the granuloma, and most importantly, capacity to support an IFNγ-recall response from newly-recruited, mycobacteria-specific CD4^+^ T cells in the granuloma and generate an IFNγ response from naïve CD4^+^ T cells *ex vivo*. Our data suggests that CD11c^+^ cells in chronic granulomas shield the bacteria locally from IFNγ-producing CD4^+^ T cells and that this mechanism involves the PD-1:PD-L signaling pathway. This protective shield provided by local CD11c^+^ cells may have contributed to the evolutionary adaptation of other infectious agents to survive within granulomatous lesions.

## Results

### Inflammatory DCs are recruited into both acute and chronic mycobacterium-induced granulomas

**Figure 1:**
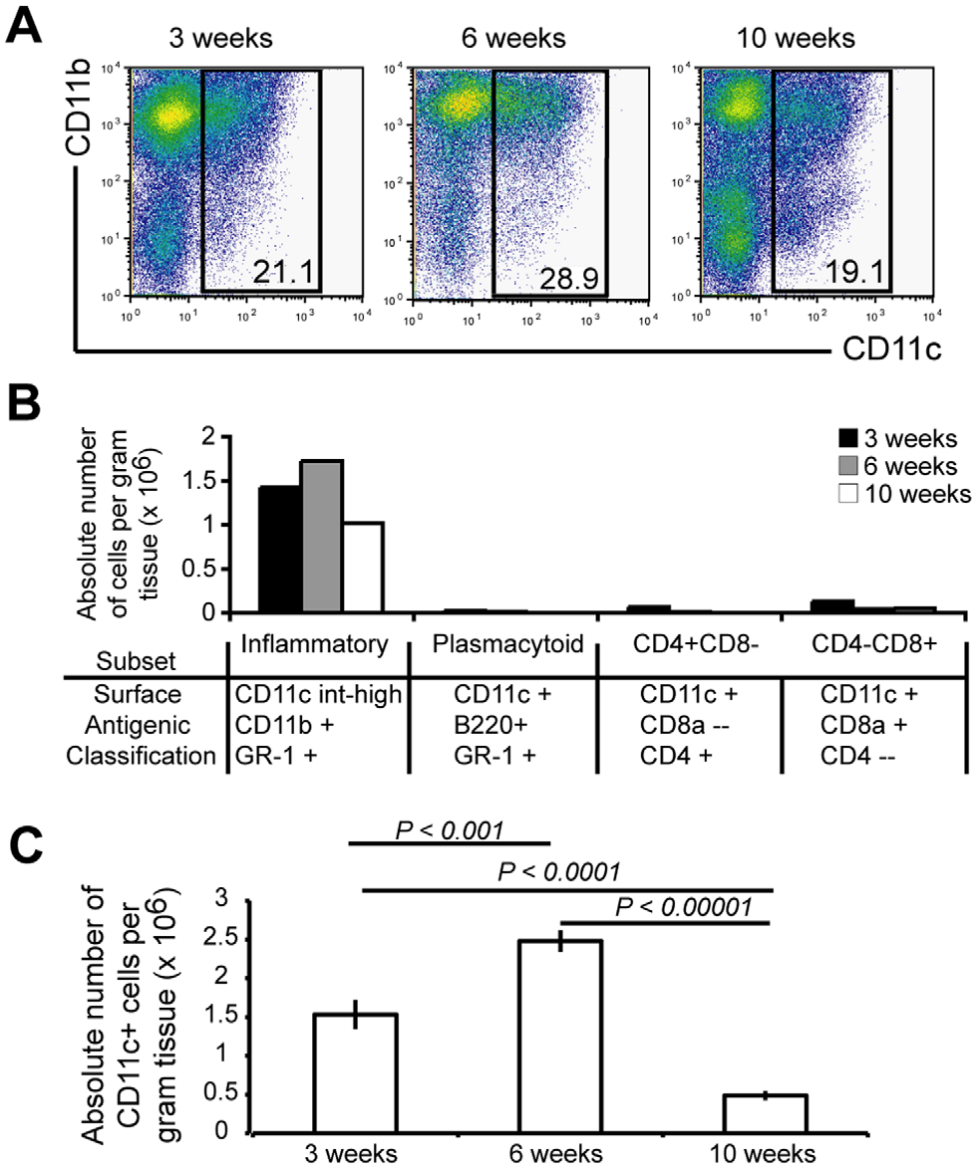
Inflammatory DCs present in both acute and chronic mycobacterium-induced granulomas. C57BL/6 mice were systemically infected intraperitoneally with BCG. Three, six and ten weeks post infection livers were harvested, and granuloma-infiltrating cells were isolated. A, FACS analysis of CD11c and CD11b populations in liver granulomas. Plot obtained from gate of SSC high FSC high population, excluding lymphocytes. B, Phenotypic analysis of CD11c^+^ cells from gating shown in A. Graph shows absolute number of cells per gram of liver tissue of cells positive for designated DC subsets listed below graph. C, Absolute number of CD11c^+^ cells per gram of tissue, population obtained from gate shown in A. Statistical differences among groups shown on graph. FACS plots and graphs generated from 3–7 mice pooled per time point for granuloma preparation and representative of three independent experiments.

Though there is an ever-growing appreciation of DCs' ability to bridge innate and adaptive immunity, little is still known about their role in chronic infection. At three, six and ten weeks after infection with BCG, granuloma-infiltrating cells were isolated from liver as previously described [29], and an isolated granuloma cell suspension was generated from 3–7 pooled mice per time point. Phenotypic analysis by flow cytometry revealed the presence of a CD11c^+^ population in both acute and chronic granulomas (Fig. 1A). Notably, the proportion of CD11c^+^ cells was similar in both three-week (acute) and ten-week (chronic) lesions. Analysis of the CD11c^+^ population showed that the predominant population in both acute and chronic lesions was the monocyte-derived “inflammatory” DC subset, characterized by CD11c^int-hi^CD11b^hi^GR-1^+^ (Fig. 1B) [30], [31]. The differentiation of this population from recruited blood-derived monocytes into inflamed tissue is well described. The frequency of this DC population within the granuloma was relatively consistent throughout infection, as measured by their gated percentage of total granulomas cells. When the absolute number of CD11c^+^cells per gram of liver tissue was determined, the significant majority of CD11c^+^ cells were present at six weeks after infection (Fig. 1C). The decreased number of CD11c^+^ cells per gram of liver tissue observed at ten weeks is likely due to the decline in both bacterial burden and subsequent number of lesions observed during chronic time points. Overall, these data demonstrate the presence of a DC-like population of cells in both acute and chronic mycobacterium-induced granulomas.

### Location of CD11c^+^ cells in granulomas changes with progression from acute to chronic infection

**Figure 2:**
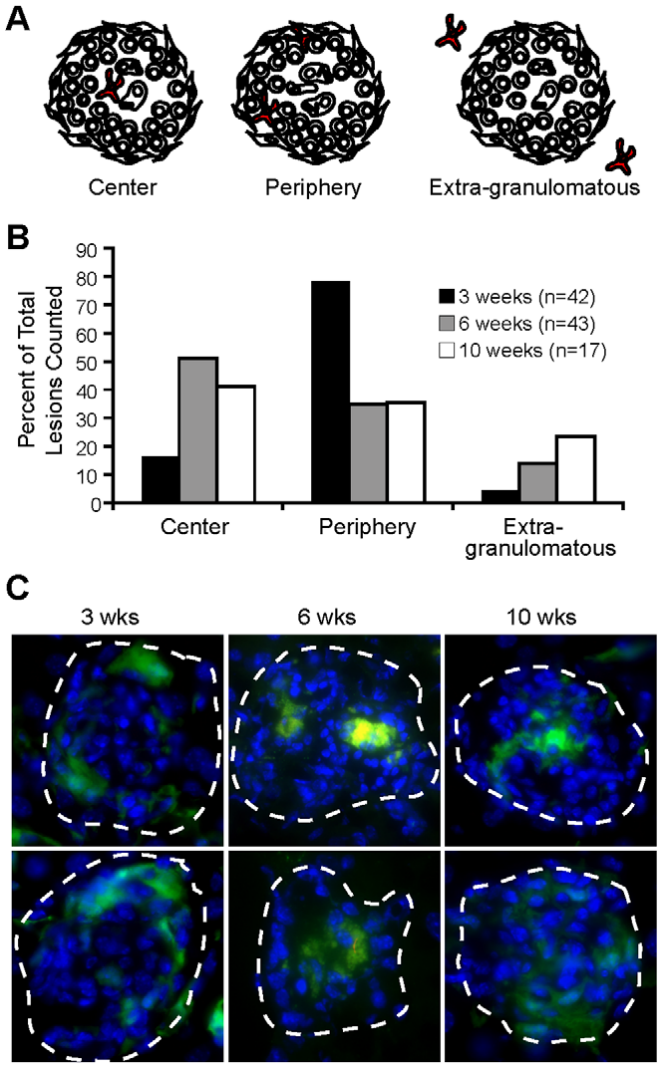
Location of DCs in acute and chronic mycobacterium-granulomas is different. A, Schematic outline of DCs in the center, periphery or outside of the granuloma. B, Distribution of DC location from stained sections. 42, 43 and 17 lesions combined from three mice per time point were observed for CD11c^+^ location for three, six and ten weeks, respectively. C, CD11c-EYFP mice were systemically infected with dsRED BCG for three, six and ten weeks. EYFP expression (green cells) and DAPI nuclear stain (blue). Granulomas are outlined with white dashed lines and shown at 1000× magnification.

The granuloma is a microenvironment with complexity in architecture and cellular distribution. Different locations within the granuloma represent diverse immunological niches for optimal interactions between antigen presenting cells, T and B cells, cytokines, and chemokines [32], [33]. As critical immune modulators, we expect the phenotype of DCs and their location in the granuloma to be important components of their function and interaction with other immune cells. To locate DCs in granulomas, liver from mice systemically infected with BCG was frozen and examined by immunofluorescent microscopy with anti-CD11c staining three, six and ten weeks after infection. We classified DC location in the granuloma based on three generalized areas: the center, the lymphoid cuff of cells forming the periphery, or outside in extra-granulomatous tissue (Fig. 2A). We observed differential CD11c^+^ cellular location throughout infection. Most CD11c^+^ cells in three-week granulomas were located in the lymphoid cuff, but as infection progressed the majority were distributed near the center of the granuloma (Fig. 2B), a common location for bacteria during chronic infection. Interestingly, CD11c^+^ cells from ten-week granulomas were distributed more evenly in all locations. We confirmed these findings using CD11c enhanced yellow fluorescent protein (CD11c-EYFP) mice with ubiquitously fluorescing DCs [34], infected with BCG -liver was analyzed by microscopy three, six and ten weeks later (Fig. 2C). Using both anti-CD11c antibody staining on tissue from WT mice and transgenic CD11c-EYFP mice, we show that this cellular subset is differently located throughout the course of infection.

### A portion of CD11c^+^ cells are infected with mycobacteria in both acute and chronic granulomas

**Figure 3:**
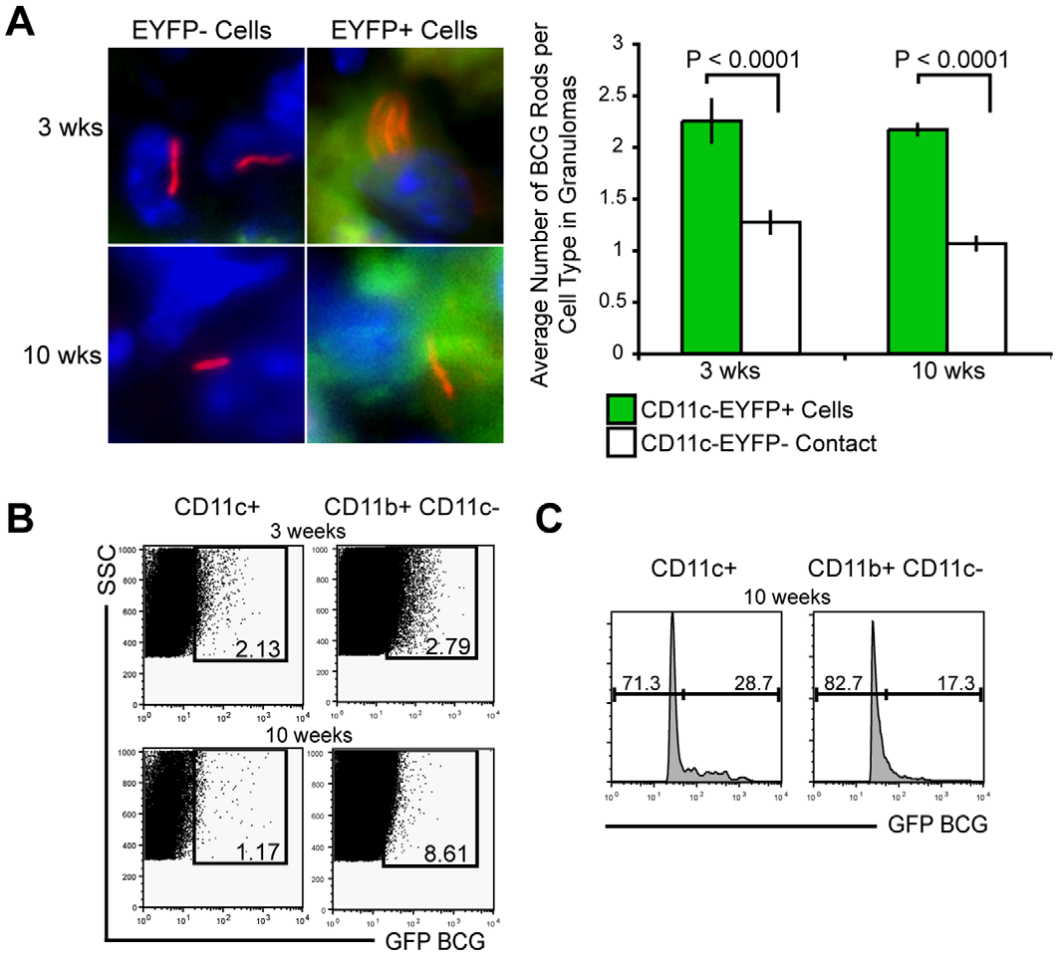
A portion of BCG is sustained within CD11c^+^ cells, both in acute and chronic granulomas. A, *Left*, Fluorescent images of CD11c-EYFP^−^ (left panels) and CD11c-EYFP^+^ cells (right panels) with dsRED BCG at three and ten weeks. Images magnified from 1000× images. DAPI nuclear stain (blue), dsRED BCG (red rods) and cytoplasmic CD11c-EYFP cells (green). *Right*, Average number of viable dsRED BCG rods per cell type (CD11c-EYFP+ or non-fluorescent) within the granuloma at three- and ten-weeks after infection. Data are represented as mean +/− SEM, *P<0.0001*. B, FACS plots generated from CD11c^+^ gate (left plot) and CD11b^+^CD11c^−^ gate (right plot). Boxed gate shows percentage of cells containing GFP-BCG. Gate placement was made from non-fluorescent-BCG infected CD11b+ and CD11c+ cells, *not shown*. Plots representative of 3- and 10-week time points with 3, 8 and 8 mice per group, respectively. C, Histograms generated from 10 week CD11c^+^ (1.17) and CD11b^+^CD11c^−^ (8.61) GFP BCG+ gates in (B).

Previous studies have shown that both resident and recruited DCs take up live bacilli, where they can persist for at least two weeks after infection [12]. Though mycobacteria infect both macrophages and DCs, activated DCs lack the ability to kill intracellular bacilli as effectively [35]. Thus, granuloma DCs may provide a long-term reservoir for bacteria during chronic infection. To investigate this possibility, we analyzed three- and ten-week granulomas from CD11c-EYFP mice infected with dsRED BCG (Fig. 3A). In both acute and chronic granulomas some BCG was located in CD11c-EYFP^+^ cells, detected by red rods located in homogenous green cytoplasmic fluorescence. Although the expected majority of bacteria were in macrophage-like cells (CD11c-EYFP^−^), there was significantly more BCG per cell in those CD11c-EYFP^+^ cells that were infected compared to than non-DCs (CD11c-EYFP^−^) both in acute and chronic lesions (Fig. 3A *Right*). Flow cytometric analysis of granuloma CD11b^+^ and CD11c^+^ cells from mice systemically infected with GFP-BCG further confirmed that both DC-like cells (CD11c^+^) and macrophage-like cells (CD11c^−^CD11b^+^) are infected at acute and chronic time points (Fig. 3B). Although the CD11c^−^CD11b^+^ cell population had a higher frequency of GFP BCG-infected cells at 10 weeks (8.61% compared to 1.17% of CD11c^+^ cells) (Fig. 3B), the 1.17% of CD11c^+^ cells infected had a higher GFP fluorescence intensity than CD11c^−^CD11b^+^ cells, 28.7% compared to 17.3%, respectively (Fig. 3C). The higher frequency of GFP intensity indicates more GFP^+^ bacilli per cell. These data suggest that while macrophage-like cells (CD11c^−^CD11b^+^) are more frequently infected, DC-like cells (CD11c^+^), when infected, contain more bacteria per cell.

### CD11c^+^ cells in chronic granulomas display decreased expression of MHCII and T cell co-stimulatory molecules, and increased expression of inhibitory molecules

**Figure 4:**
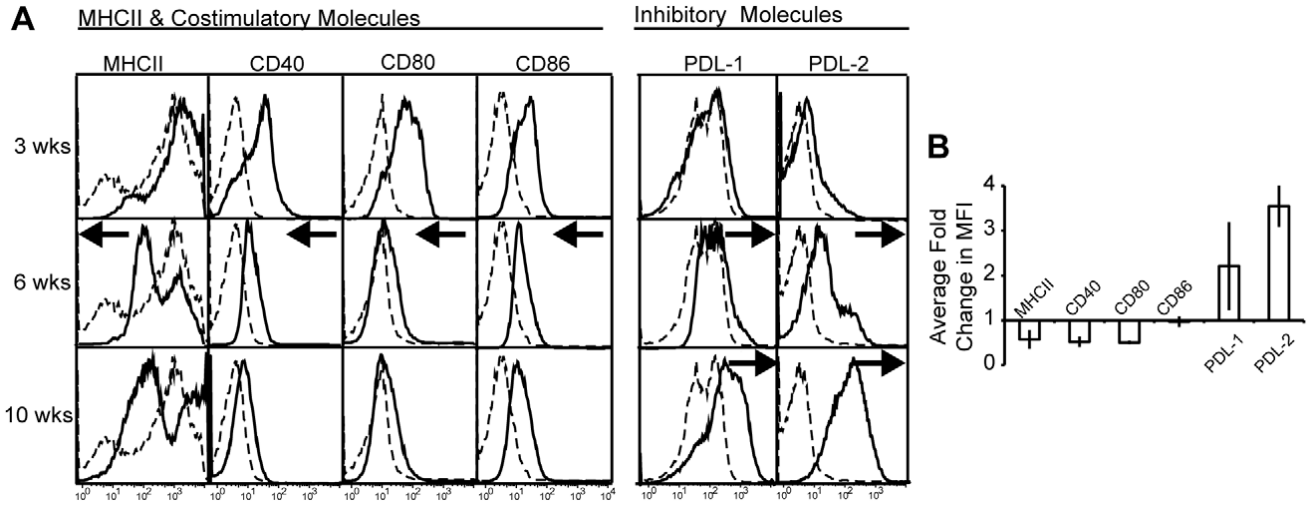
Surface expression of activating and inhibitory costimulatory molecules and chemokine receptors is different on CD11c^+^ cells in acute and chronic granulomas. CD11c^+^ cells from granuloma single cell suspensions obtained from three, six and ten week systemically infected C57BL/6 mice were phenotyped using flow cytometry. A, Histograms represent fluorochrome surface staining with monoclonal antibodies against MHCII, activating costimulatory molecules (CD40, CD80 and CD86) and inhibitory costimulatory molecules (PD-L1 and PD-L2). Black-dashed histograms represent costimulatory molecule expression on naïve splenic CD11c^+^CD11b^+^ cells. Blacks arrows indicate directional shift in MHCII and costimulatory molecule expression compared to 3-week expression levels at time points where substantial change is observed. Histograms representative of 3 independent experiments with 3–7 mice per group. B, Average fold change between three and ten week time points in mean fluorescent intensity of MHCII and costimulatory molecules. Average generated from fold change in three independent experiments.

DCs are equipped with an array of receptors that facilitate T cell activation. To investigate the phenotype of DCs in granulomas throughout infection, wild-type mice were infected with BCG and granuloma-infiltrating cells were isolated at three, six and ten weeks after infection. Granuloma cells were stained with antibodies against MHCII, costimulatory (CD40, CD80 and CD86) and inhibitory (PD-L1 and PD-L2) surface molecules and analyzed by flow cytometry (Fig. 4A). After gating on CD11c^+^ cells (Fig. 1A), we observed a distinct change in phenotype of this cellular subset from three weeks to ten weeks after infection. Compared to naïve splenic CD11c^+^ cells, CD11c^+^ cells from three-week granulomas had high expression of MHCII, T cell costimulatory molecules, CD40, CD80 and CD86, and low expression of inhibitory molecules PD-L1 and PD-L2. Ten weeks after infection these cells had lower expression of T cell costimulatory molecules, and higher expression of both PD-L1 and PD-L2 (Fig. 4A Black arrows indicate directional shift of MHCII and costimulatory molecule expression compared to expression level observed 3 weeks after infection). MHCII and costimulatory molecules CD40, CD80, and CD86 had an average 0.5 fold decrease in their MFI intensity from three to ten weeks after infection (Fig. 4B). In contrast, PD-L1 and PD-L2 increased in their mean MFI by 2 and 3.5 fold, respectively. These data suggests that DC-like cells from early three-week granulomas would support potent T cell activation (or re-activation), but those from 10 week granulomas may not.

### The majority of CD4^+^ cells maintain close contact with CD11c-EYFP^+^ cells in both early and chronic granulomas

**Figure 5:**
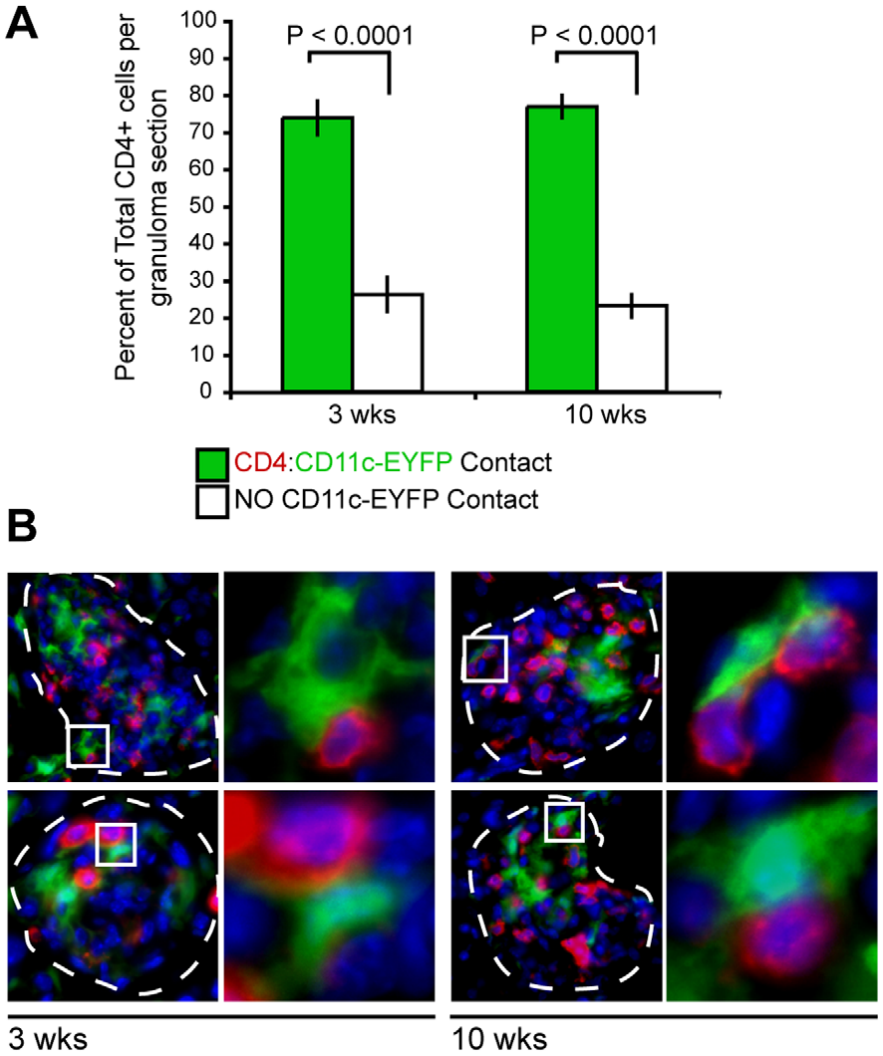
CD4^+^ T cells maintain contact with CD11c-EYFP^+^ cells in both acute and chronic granulomas. A, Quantitative analysis of CD4^+^ T cell contact with CD11c-EYFP^+^ cells in granulomas from three- and ten-week liver sections. All CD4^+^ T cells per granuloma section were determined to be in contact (green bars), or not in contact (white bars) with CD11c-EYFP^+^ cells. For each time point, liver sections from 3 independent mice were used, and 10–15 lesions per section were analyzed. Data are represented as mean +/− SEM, *P<0.0001*. B, Fluorescent images demonstrating CD11c-EYFP contact with CD4^+^ cells. Granulomas are outlined with white dashed lines at 1000× magnification and colors shown depict CD11c-EYFP (green cells), anti-CD4 Alexa 647 (red cells), and DAPI nuclear stain (blue).

The differential expression of costimulatory and inhibitory molecules on acute and chronic CD11c^+^ cells likely has different consequences on T cell responsiveness in acute and chronic granulomas. We have already shown that the location of this cellular subset changes in the granuloma throughout infection (Fig. 2). Next, we investigated if the differential location of CD11c^+^ cells in acute and chronic granulomas changed the frequency of contact between T cells. We speculated that changes in DC phenotype would be less relevant to T cell function if contact between the two cell types was ablated by the altered DC location in the lesion. We also speculated that any loss of contact between the two cell types would result in an inability of DCs to re-stimulate newly primed T cells recruited to granulomas. To address this question, we quantitatively analyzed three- and ten-week granulomas for CD4^+^:CD11c-EYFP^+^ cellular contact by fluorescent immunohistochemistry (Fig. 5A and B). This was done by staining liver sections from three- and ten-week infected CD11c-EYFP mice with fluorochrome-labeled anti-CD4, and analyzing all CD4+ cells per lesion. We observed similar frequencies of CD4^+^:CD11c-EYFP^+^ cell contact between three- and ten-week granulomas, and importantly, found that the statistically significant majority of CD4^+^ T cells from both three- and ten-week granulomas were in contact with CD11c^+^ cells. Under high magnification, co-localization of CD11c-EYPF^+^ (DCs) and dsRed (CD4^+^ T cells) is easily seen (Fig. 5B- 2^nd^ and 4^th^ column, 1^st^ and 2^nd^ row). The few CD4^+^ T cells that were not in contact with CD11c^+^ cells may be due to restrictions of the sectioned plane, such that contact with a CD11c^+^ cell may be above or below the plane. These data demonstrate that CD11c^+^ cells maintain physical contact with CD4^+^ T cells in both acute and chronic granulomas. Collectively, these data, in combination with the observed phenotypic changes (Fig. 4), suggest that DC-like cells may have direct effects on both resident and newly recruited mycobacterium-specific T cells during both early and chronic infection.

### CD4^+^ T cells produce less IFNγ in chronic compared to acute granulomas

**Figure 6:**
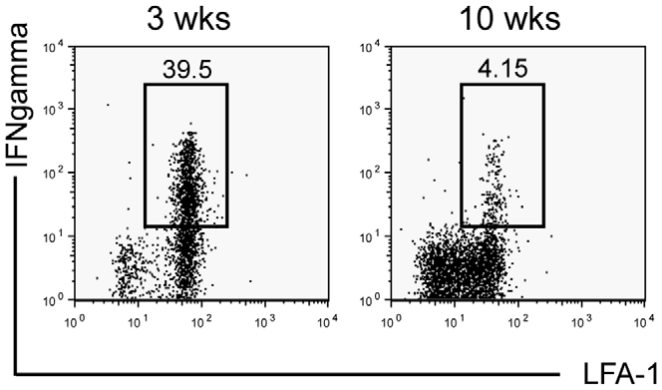
IFNγ recall response of CD4^+^ T cells is much higher in acute compared to chronic granulomas. Granuloma cells from three- and ten-week systemic BCG infected animals were isolated and stimulated *ex vivo* for five hours with anti**-**CD3. FACS plots show intracellular IFNγ staining and LFA-1 expression from CD4^+^ gated population. Plots representative of five pooled mice per group.

To test for differences between acute and chronic granuloma-resident CD4^+^ T cell activity, granuloma cells from both lesions were re-stimulated *ex vivo* after systemic infection with BCG. CD4^+^ T cells from ten-week granulomas showed a dramatic decrease in both IFNγ production and LFA-1 expression compared to three-week granulomas (Fig. 6). In fact, the IFNγ-producing T cell population in ten-week granulomas fell below our detection level in many samples. These observations, along with those detailing an inhibitory phenotype of CD11c^+^ cells in chronic granulomas suggests a suppressive DC population in chronic granulomas. Since many current therapeutic efforts are aimed at generating an increase in Mtb-specific IFNγ-producing Th1 cells, we next tested the ability of DCs to re-boost newly recruited CD4^+^ T cells in acute and chronic granulomas.

### APCs in chronic granulomas support lower reactivation of newly recruited mycobacterium-specific IFNγ-producing CD4^+^ cells compared to acute granulomas

**Figure 7:**
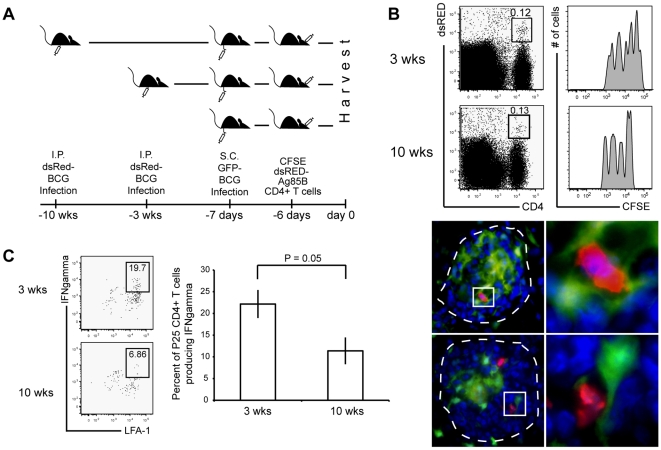
Newly activated Ag85B-specific Th1 cells are recruited at similar levels to acute and chronic granulomas, but produce less IFNγ in chronic granulomas. A, Experimental schema. Coinciding three- and ten-week dsRED BCG systemically infected CD11c-EYFP mice received s.c. footpad injections of 1.25×10^6^ CFU GFP BCG seven days prior to harvest. The following day 10^6^ CFSE dsRED Ag85B-specifc transgenic CD4^+^ T cells were adoptively transferred via retro-orbital sinus vein injection. Mice were harvested six days later and granuloma cells were isolated. B, Left FACS panel, Presence of transferred P25 dsRED CD4^+^ T cells in both three- and ten-week granulomas. CD4^+^dsRed^+^ co-staining indicates adoptively transferred population. Plots derived from lymphocyte gate on SSC vs. FSC. B, Right FACS panel, CFSE dilution of gated P25 dsRED CD4^+^ T cell population indicates the recent activation of these T cells. B, Lower panels, Fluorescent microscopy reveals contact between CD11c-EYFP cells (green) and P25 dsRED CD4^+^ T cells (red) in granuloma (DAPI-blue). Images taken at 1000× magnification. Right images, Enlarged image of small white box. C, Left FACS panels, *ex vivo* restimulation of granuloma cells and staining for LFA-1 expression and IFNγ production. Plot was obtained from gated dsRED CD4^+^ T cell population as shown in B. C, Right graph, Average P25 IFNγ production in the granuloma from three independent experiments, displayed as a percent of total CD4^+^dsRED/Vβ11^+^ cells in lymphocyte gate (data are represented as mean +/− SEM). Plots representative of three independent experiments with 2–7 mice per group. These data show that despite CD4^+^IFNγ-producing mycobacterium-specific T cells reaching both acute and chronic granulomas, the IFNγ response is blocked (not reactivated) in chronic lesions.

**Figure 8:**
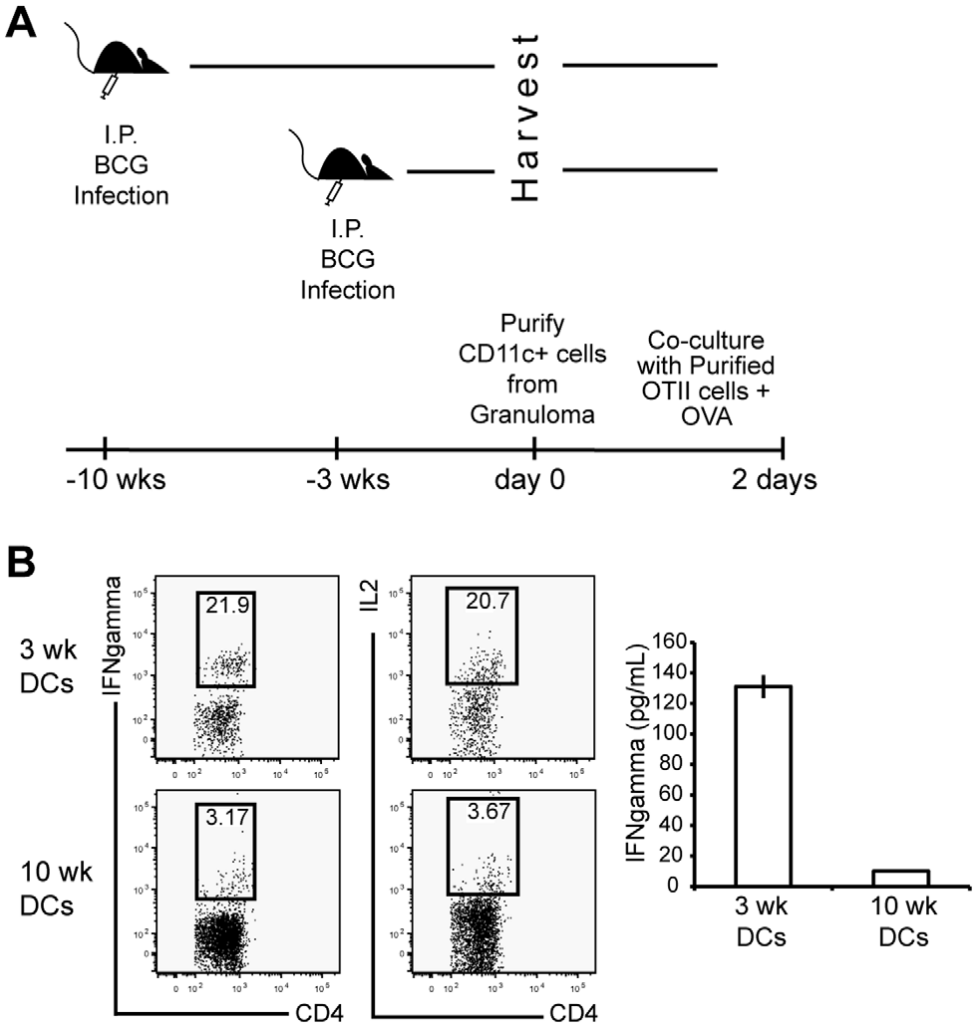
Naïve CD4^+^ OTII T cell activation by CD11c^+^ cells from acute and chronic granulomas. A, Experimental schema. B, CD11c^+^ cells from three- and ten-week BCG infected animals were purified and cultured with purified naïve OTII CD4^+^ T cells at 5∶1 ratio of T cell reponders:APCs, respectively. Cells were co-cultured *ex vivo* for 48 hours, followed by a five hour recall with anti-CD3. Cells were stained for intracellular cytokines IFNγ (left FACS plots) and IL2 (right FACS plots). B right graph, IFNγ ELISA of supernatant following 48 hours *ex vivo* co-culture. Cells shown were from CD4^+^Vβ5^+^ lymphocyte gate. Granuloma cells from 5–8 mice were pooled for each time point.

A recent study by Egen and colleagues demonstrated the movement of T cells in granulomas, and in doing so, also demonstrated their ability to interact with many local cells [17]. Additionally, newly primed T cells require a second antigen encounter at the site of inflammation in order to rapidly secrete cytokines [36]. Here, we tested the ability of granuloma-resident APCs from three- and ten-week granulomas to induce IFNγ production from newly recruited mycobacterium-specific T cells that migrated to pre-existing granulomas. For this, CD11c-EYFP mice were infected with dsRED BCG followed three or ten weeks later by adoptive transfer of CFSE-labeled, dsRED CD4^+^ T cells specific for mycobacteria-secreted antigen 85B (Fig. 7A). One day before adoptive transfer of Ag85B-specific T cells, mice received subcutaneous footpad injections of GFP-BCG to provide an immune boost. GFP-BCG was used for the secondary systemic boost in order to distinguish between previously established three and ten-week dsRED BCG-induced granulomas in the liver. We expected this immune boost to induce migration of adoptively transferred cells to the draining popliteal lymph nodes and acquire a Th1 phenotype, mimicking a therapeutic boost intended to generate a high systemic level of mycobacterium-specific IFNγ-producing CD4^+^ T cells. Six days after adoptive transfer, cells from three- and ten-week granulomas were isolated for *ex vivo* recall of IFNγ production. CD4^+^dsRED^+^ co-staining identified adoptively transferred 85B antigen-specific TCR transgenic T cells. Analysis by flow cytometry showed that the proportion of LFA-expressing, adoptively transferred T cells that migrate into three- and ten-week granulomas is similar, demonstrating that T cell recruitment into chronic granulomas was not hindered (Fig. 7B). Fluorescent microscopy confirmed the presence of newly recruited Ag85B-specifc T cells into the granulomas and also showed their proximity to CD11c-EYFP cells (Fig. 8B, lower panel). Importantly, upon *ex vivo* stimulation, there was a lower frequency of adoptively transferred Ag85-specific T cells in chronic granulomas that were able to produce IFNγ compared to three-week granulomas (Fig. 7C individual FACS plots). These data support the hypothesis that DC-like cells in chronic lesions, which express high levels of inhibitory molecules, suppress local IFNγ production from newly recruited mycobacterium-specific T cells. These experiments are relevant because they attempt to evaluate the potential effectiveness of current vaccine and therapeutic efforts aimed at increasing the activity and number of IFNγ-producing CD4^+^ T cells. Therefore, these data suggest that despite a high level of systemic mycobacterium-specific IFNγ-producing T cells, DCs may be shielding bacteria within the granuloma environment from IFNγ, a critical component for controlling infection.

### CD11c^+^ cells in chronic granulomas are less efficient at inducing an IFNγ-producing CD4^+^ T cell immune response

Our data suggests that CD11c^+^CD11b^+^ cells in chronic granulomas have an inhibitory phenotype (Fig. 4) and down regulate systemic IFNγ producing T cells after they home to granulomatous lesions (Fig. 7). The fact that chronic granulomas contain much less bacterial antigen might provide an alternative explanation to the previous observation of limited IFNγproduction in chronic lesions. To test this possibility, CD11c^+^ cells were purified from three- and ten- week granulomas and co-cultured with purified, naïve OT-II CD4^+^ T cells (Fig. 8A). Exogenous OVA protein alone was added to cultures, 48 hours later cells were analyzed for IFNγ and IL-2 production by flow cytometry (Fig. 8B). CD11c^+^ cells purified from three-week granulomas were more efficient at inducing IFNγ and IL-2 production from OT-II CD4^+^ T-cells than CD11c^+^ cells from ten-week granulomas. IFNγ production was confirmed by ELISA after *ex vivo* stimulation for 48 hours (Fig. 8B Right). These data show that even though differential amounts of antigen in acute and chronic granulomas may contribute to the decreased efficiency of mycobacterium-specific T cells to produce IFNγ, DCs from acute and chronic granulomas have an inherent difference when regulating local IFNγ responses. Furthermore, these data collectively suggest that an inhibitory population of CD11c^+^ cells in chronic granulomas down-regulates the activity of IFNγ-producing CD4^+^ T-cells.

### Blockade of PD-L:PD-1 signaling increases the ability of CD11c^+^ cells in chronic granuloma to reactivate CD4^+^ T cells *ex vivo*

**Figure 9:**
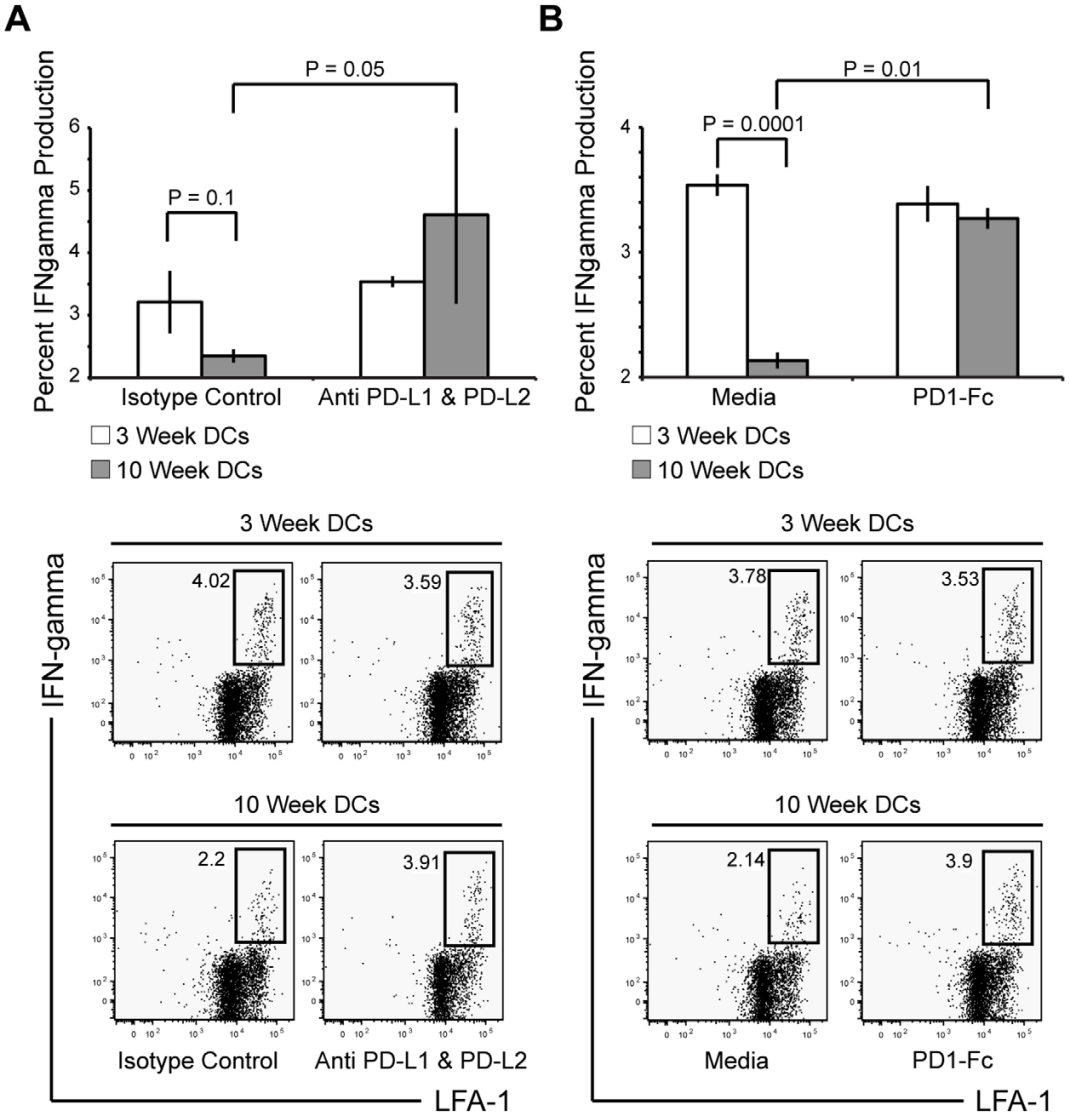
Blockade with anti-PD-L1 and anti-PD-L2 mAbs, or PD1-Fc restores 10-week granuloma CD11c^+^ cells' ability to restimulate allogenic CD4^+^ T cells to produce IFNγ. DCs (2×10^4^) purified from 3- and 10-week granulomas were cocultured *ex vivo* with purified, primed Ag85B-specifc transgenic CD4^+^ T cells (1×10^5^). Intracellular IFNγ production was measured four hours after DC pretreatment with isotype control or anti-PD-L1 and anti-PD-L2 pretreatment (A), and media or PD1-Fc pretreatment (B). FACS lots were derived from initial lymphocyte gate on SSC vs. FSC, followed by gating of CD4^+^Vβ11^+^ population. Representative FACs plots are shown from groups of 3–7 mice per group. Data are represented as mean +/− SEM.

Previous studies have demonstrated that blockade of the PD-L:PD-1 pathway enhances T cell responses [37]–[40]. To determine if increased PD-L expression shown on CD11c^+^ cells from chronic granulomas (Fig. 4) had a direct effect on their inability to stimulate CD4^+^ T cells activation (Fig. 7 and 8), we tested the effect of CD11c^+^ PD-L- T cell PD-1 interaction on T cell responses. CD11c^+^ cells were purified from three- and ten-week granuloma preparations by magnetic separation and were pretreated with media or isotype controls, 10 µg/mL of both anti-PD-L1 and -PD-L2 mAbs, or 50 µg/mL of soluble PD1-Fc that binds both PD-L1 and PD-L2 [41]. After 1 hour, purified Ag85B-specific CD4^+^ T cells from lymph nodes of 3 week BCG-infected P25 mice were added to the DCs at a ratio of 5∶1 (CD4^+^:CD11c^+^). Protein transport inhibitor and 0.5 µg/mL Ag85B peptide was added to each coculture, and intracellular IFNγ was measured after 4 hours for flow cytometry. Treatment with anti-PD-L1 and -PD-L2 mAbs (Fig. 9A), and PD1-Fc (Fig. 9B) significantly increased IFNγ production from T cells stimulated by CD11c^+^ cells from 10-week granuloma. Both PD-L blocking treatments restored IFNγ to production levels achieved by 3-week CD11c^+^ cell stimulation. Collectively, these data suggest that DCs in chronic granulomas actively inhibit antigen-specific T cell responses in a way that facilitates chronic infection and survival of *Mycobacterium bovis*.

## Discussion

Here we report that a DC-like population, characterized by CD11c^+^ expression, are present in acute and chronic BCG-induced granulomas, but their location, phenotype, and ability to induce IFNγ responses distinctly changes throughout the course of infection. CD11c^+^ cells in acute lesions are primarily located in the lymphoid cuff of the granuloma and have high expression of MHCII and T cell stimulatory molecules CD40, CD80 and CD86. However, those from chronic granulomas have low expression of MHCII and co-stimulatory molecules, high expression of T cell inhibitory molecules PD-L1 and PD-L2, and also migrate toward the granuloma center. Most importantly, CD11c^+^ cells from acute granulomas have a greater capacity to induce IFNγ production from CD4^+^ T-cells compared to those from chronic granulomas. When PD-L expression on CD11c^+^ cells was blocked *ex vivo*, their ability to restimulate allogenic CD4^+^ T cells to produce IFNγ was restored to the level observed during acute infection. Although these data demonstrate a direct effect of granuloma DCs on CD4+ T cell IFNγ production *ex vivo*, they do not rule out the possibility that other factors within the granuloma may also influence IFNγ production. This CD11c^+^ shield may induce local tolerance within chronic granulomas, despite the high systemic level of CD4^+^IFNγ^+^ anti-mycobacterial T cells. The ability of CD11c^+^ cells to shield mycobacteria from local IFNγ T cell responses may be one explanation for the ability of mycobacteria to survive indefinitely in granulomas.

IFNγ is critical for activation of mechanisms in macrophages that kill engulfed bacteria –these include production of reactive nitrogen and oxygen species, as well as stimulation of phagosome-lysosome fusion [2]. The necessity of mycobacterium-specific IFNγ-producing CD4^+^ T cells for controlling mycobacterial growth is demonstrated in mouse models with ablated CD4^+^ T-cell responses, HIV infected individuals, and patients lacking functional IFNγ receptors, all of which are unable to control Mtb infection [4], [42]–[47]. While it seems rational that improved vaccine strategies have aimed to increase CD4^+^ T-cell responses, the results have unfortunately been ineffective at achieving bacterial clearance [48], [49]. Our work here detailing the presence of an inhibitory DC-like population in chronic granulomas provides a possible explanation for these failures.

PD-Ls have been shown to negatively regulate T cells by inhibiting proliferation and cytokine production and are exploited by several microorganisms to evade anti-microbial immunity [50], [51]. PD-L expression on antigen presenting cells is also key for the generation of peripheral tolerance [52]. What drives the transition of a population of DCs that stimulate T cells to one that inhibits them is a key question. One possibility is that the proinflammatory environment of acute granulomas provides signals to drive increased expression of PD-L receptors on APCs. IFNγ itself, which is abundant in acute granulomas, up-regulates PD-L on DCs [41], [51], [53]–[55]. To test potential IFNγ regulation of PD-L expression on DCs during BCG infection, we infected IFNγ KO mice, but observed no differences in PD-L expression compared to wild-type mice six weeks after infection (*data not shown*). However, this is likely due to compensatory effects of other proinflammatory factors in the granuloma, such as TNFα, IL-6, IL-1β, IL-4, GM-CSF, and/or TSLP, which have all been found to induce an inhibitory DC phenotype [56], [57]. In addition to host-derived products, certain mycobacterial antigens have also been shown to interfere with DC maturation and down-regulate the ability of the DCs to induce Th1-IFNγ responses from antigen-specific T cells [58]–[61]. TLR 2 and 4 stimulation has been shown to induce inhibitory and tolerogenic DCs, while PD-L up-regulation results from TLR 7/8 stimulation [56], [62].

In accordance with our finding that CD11c^+^ cells in human Mtb-induced granulomas express PD-L1 and -2 *(data not shown)*, recent data by Juardo *et al.* shows that mycobacterial antigen-induced expression of PD-1 on lymphocytes from patients with tuberculosis interferes with anti-mycobacterial T cell effector functions [40]. PD-L1 expression has also been shown in BCG-induced bladder granulomas from patients receiving BCG therapy for urothelial carcinoma of the bladder [63]. Together with our own data, these human data suggest that once lymphocytes reach Mtb-induced granulomas, PD-1: PD-L1/2 ligation may interfere with effector T cell responses. A recent finding by Einarsdottir et al. has shown that administration of anti-PD-1 and -PD-L1 blocking antibodies at the onset of chronic *Mycobacterium tuberculosis* infection in mice does not effect CD8+ T cell function or bacterial load four weeks later [71]. It is difficult to compare data from *in vivo* and *in vitro* experiments, but one possible explanation for the lack of response observed *in vivo* is that PD-L2 was not also selectively blocked in their study. PD-L2 is more exclusively expressed by DCs and can also ligate with PD-1, potentially compensating for any blocking of PD-L1 [41].

DCs may also contribute to latency by acting as a long-term reservoir for mycobacteria, ensuring their survival in highly bactericidal environments like the granuloma. Not only does our data show BCG-infected CD11c^+^ cells in acute and chronic granulomas, but those CD11c^+^ cells infected often contained more than one bacterium per cell. It has been reported that DCs are less efficient at killing intracellular mycobacteria [35], [64]–[66], though altered compartmental trafficking of bacilli inside DCs prohibits mycobacterial access to the necessary nutrients required for growth [67], [68]. These studies ultimately suggest two things. The first is that while the majority of mycobacteria infect macrophages, a portion of mycobacteria that infect DCs may avoid the effective killing mechanisms of macrophages. The second is that mycobacterium may infect DCs so that they can influence the immunosuppressive effects of the PD-L1/2 system. Studies have shown that human monocyte-derived DCs infected with either Mtb or BCG have reduced expression of MHCII and CD80, produced IL-10 over IL-12, and had an impaired ability to generate IFNγ-secreting CD4^+^ T cells [69]–[72]. Furthermore, a recent *in vivo* study by Wolf *et al.* showed that the myeloid DC population responsible for shuttling Mtb from the lungs to the draining lymph nodes in mice poorly stimulated IFNγ production from Ag85B-specific CD4^+^ T cells [15]. While these data suggest that mycobacteria-infected DCs induce immunosuppressive effects, a body of data actually suggests the opposite. Upon infection of both murine and peripheral monocyte-derived human DCs with either Mtb or BCG *in vitro* and *in vivo*, DCs matured, produced Th1-promoting cytokines, and induced IFNγ-producing T cells [73]–[79]. This collection of conflicting data may be the result of the various models used, but taken together; the data collectively demonstrate the multi-functionality of DCs during infection.

**Figure 10:**
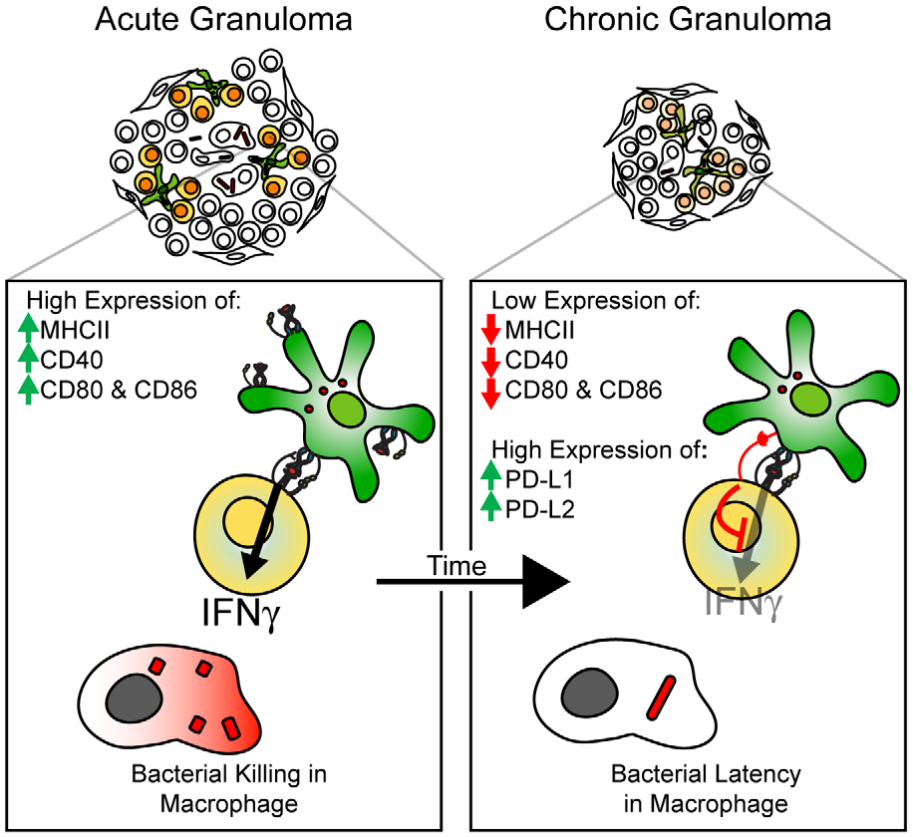
Changing role of dendritic cells in mycobacterium-induced granulomas over time. Scheme showing the changing phenotype of granuloma DCs in the transition from acute to chronic infection. Acute lesions contain DCs with high MHCII, and T cell costimulatory molecule (MHCII, CD40, CD80/86) expression. As the infection becomes chronic, DCs within the granuloma have decreased expression of MHCII, activating costimulatory molecules, and increased expression of inhibitory molecules PD-L1 and PD-L2. Expression of these inhibitory molecules results in an inability of local CD11c^+^ cells to reactivate IFNγ-producing CD4^+^ T cells, and therefore, shielding the granuloma-located bacteria from an important anti-bacterial factor.

The scheme in Figure 10 summarizes our results, which indicates a distinct change in CD11c^+^ cell phenotype and function in bacteria-containing granulomas (Fig. 10). It is possible that DCs, by affecting local IFNγ availability, may contribute to granuloma maturation and bacterial persistence. Future studies of DC function in chronic Mtb-induced granulomas will be needed to both extend our findings and develop vaccine strategies that would weaken the shielding effect of DCs in chronic granulomas.

## Materials and Methods

### Mice

Wild type C57BL/6 (H2^b^) and IFNγ-deficient mice were purchased from the Jackson Laboratory (Bar Harbor, ME). CD11c-EYFP transgenic mice on the C57BL/6 background were a generous gift from Dr. Michel C. Nussenzweig (Rockefeller University, NY) [34]. P25 transgenic mice on the Rag−/− background were a generous gift from Drs. Rothfuchs and Sher (NIH, Bethesda, MD). P25 mice were bred with Actb-DsRED.T3 transgenic mice from the Jackson Laboratory to obtain dsRED P25 mice.

### Ethics Statement

Mice were housed and bred in microisolator cages in a pathogen-free facility at the University of Wisconsin Animal Care Unit (Madison, WI), and husbandry and experimental procedures were performed according to the guidelines of the Institutional Animal Care and Use Committee. All animal protocols were approved by the University of Wisconsin Institutional Animal Care and Use Committee. Animal protocol approval under protocol M01616.

### Infection

The Pasteur strain of BCG (Staten Serum Institute, Denmark), kanamycin-resistant GFP-expressing BCG, or kanamycin-resistant dsRED-expressing BCG were grown in Middlebrook 7H9 supplemented with 0.05% Tween 80 and 10% oleic acid-dextrose-catalase supplement (Difco, Detroit, MI) in the presence or absence of kanamycin (50 µg/mL) and stored in frozen aliquots at −80°C. For infections, ampoules were thawed, diluted in PBS, and briefly sonicated to obtain single-cell suspensions. For systemic infection, a non-lethal dose of 1×10^7^ CFU in 100 µL was intraperitoneally injected. For localized infection, a dose of 1.25×10^6^ CFU was injected subcutaneously into the footpads. Kanamycin-resistant GFP-BCG was generated using an HSP60 promoter driven GFP plasmid, generously provided by Dr. Glen Fennelly (Albert Einstein University, NY), and dsRED BCG was a generous gift from Dr. Lalita Ramakrishnan (University of Washington, WA). Growth and preparation of wild-type BCG Pasteur strain (Staten Serum Institute, Copenhagen, Denmark) were performed as previously described [29].

### Mononuclear cell isolation

Aseptic isolation of splenocytes and lymph nodes was performed using standard methods. Granuloma preparations were performed as previously described [29], [80]–[82]. Briefly, granuloma suspensions were prepared from freshly excised infected livers. The livers were homogenized in a Waring tissue blender, and granulomas were allowed to settle by virtue of their higher density compared to other hepatic cells. After settling the supernatant was decanted, and granulomas were washed in RPMI 1640 medium and centrifuged at 1,500 rpm for 5 minutes. The washed granuloma pellets were digested with 5 mg/ml type I collagenase (Sigma-Aldrich, St. Louis, MO) at 37°C for 40 min with shaking. The softened granulomas were disrupted by repeated expulsion through a syringe for 1 minute. To remove debris, the suspension was filtered through a 70 µm nylon cell strainer (BD Bioscience, San Jose, CA). After lysing red blood cells, cell suspension was washed twice with RPMI 1640 medium and live leukocyte count was determined by trypan blue staining. Granuloma suspensions were obtained by pooling at least 3 or more infected livers per time point.

### Flow cytometry

A total of 10^6^ cells were incubated for 30 min on ice with saturating concentrations of labeled Abs with 40 µg/mL unlabeled 2.4G2 mAb to block binding to Fc receptors and washed 3 times with staining buffer (PBS plus 1% BSA). Fluorochrome-labeled Abs against CD11c (HL3), CD11b (Mac-1), CD4 (RM5-4), LFA-1 (2D7), B220 (RA3-6B2), MHCII I-A^b^ (AF6-120.1), CD40 (3/23), CD80 (16-10A1), CD86 (GL1), IFNγ (XMG1.2), IL-2 (JES6-5H4), and Vβ11 (RR3-15) were purchased from BD Biosciences (San Jose, CA). Fluorochrome-labeled Abs against CD8α (53-6.7), PD-L1 (MIH5), and PD-L2 (TY25) were purchased from eBioscience (San Diego, CA). Anti-CD16/CD32 (2.4G2), anti-CD3 (145–2C11) and anti-GR-1 (RB6) Abs were produced from hybridomas. For intracellular cytokine staining, single-cell suspensions from various tissues were cultured at 37°C in complete RPMI 1640 supplemented with GolgiStop (BD Biosciences) in the presence of anti-CD3 Ab for 5 h. After surface staining with anti-LFA-1 and anti-CD4 Abs, cell suspensions were fixed and permeabilized by Cytofix/Cytoperm solution (BD Biosciences), followed by staining with anti-IFNγ and/or IL-2 Ab. Cell surface staining was acquired on a FACSCalibur or LSRII (BD Biosciences, San Jose, CA) and analyzed with FlowJo (Tree Star) software version 5.4.5.

### Generation of recombinant PD1-Fc construct

PD1-Fc was generated by fusing the extracellular domain of PD1 to a human IgG1 Fc fragment. PD1 was amplified from spleen-derived cDNA using oligonucleotides specific for the signal sequence (EcoR1PD1, atc gaa ttc acc tga gat gtc ttc ttt tgc) and the membrane proximal region of the extracellular region (hingePD1, ggc atg tgt gag ttt tgt cac cag att tgg gct caa ctt tgc ctt gaa acc ggc ctt ctg g). The Fc region of human IgG1 was amplified from the vector pHC-huIgG1 [83]. PCR products of PD1 and of Fc were fused by overlap-extension PCR, where the hinge region served as the overlapping sequence. The fused product was digested by EcoRI and XhoI and ligated into the pHC-huIgG1 plasmid from which IgG1 was excised by EcoRI and XhoI. The fusion protein was purified from supernatant of transfected CHO-K cells using protein G affinity chromatography.

### OTII stimulation and P25 reactivation by DCs from acute and chronic granulomas

DCs were isolated from 3- and 10-week granuloma preparations using a CD11c MicroBeads Cell Separation Kit from Miltenyi Biotec (Auburn, CA). Live transgenic OTII CD4^+^ T cells were sorted using a FACSVantageSE at the University of Wisconsin Comprehensive Cancer Center's Flow Facility. Pooled lymph node CD4+ Ag85b-specific T cells from 3-week BCG infected were purified using a Dynal Mouse CD4 Negative Isolation Kit following manufactures protocol (Invitrogen, Carlsbad, CA). CD4^+^ T cells (1×10^5^) were co-cultured with sorted CD11c^+^ cells at a 5∶1 ratio of T cells:DCs, respectively. For OTII activation, cells were cultured in a 96 well plate in complete RPMI in the presence of 5 µg/mL OVA for 2 days at 37°C with 5% CO_2_. Frozen supernatant from 2-day culture was used for IFNγ ELISA, samples ran in quadruplicate. Ready-Set-Go! Mouse Interferon gamma ELISA kit was used following manufacture's protocol (eBioscience, San Diego, CA). For P25 reactivation, sorted DCs were pre-cultured in a 96 well plate (2×10^4^) in complete RPMI with either media, both isotype controls (Rat IgG2a κ and IgG2b κ - eBioscience, San Diego, CA), both anti-PD-L1 (10F.9G2- BioLegend, San Diego, CA) and anti-PD-L2 (TY25- eBioscience, San Diego, CA) at 10 ug/mL each, and Fc-PDL at 50 ug/mL for 1 hour prior to addition of T cells. Purified T cells (1×10^5^) were added to each well along with GolgiStop (BD Biosciences) and 0.5 ug/mL P25 peptide (Mtb Ag85B AA240–254, Invitrogen Carlsbad, CA). After 4 hours at 37°C with 5% CO_2_ cells were stained for intracellular cytokine, IFNγ, for flow cytometry analysis.

### Fluorescent Microscopy

Organs fixed over night in 3% formalin/25% sucrose in PBS were frozen down in O.C.T Compound (Tissue-Tek Sakura, Torrance, CA). 10 µm thick cryosections were cut from O.C.T-embedded liver tissue samples and fixed for 10 or 30 min in ice-cold acetone or 4% formalin in PBS, respectively, then washed three times with PBS and outlined with a Pap pen. Sections were blocked with 40 µg/mL 2.4G2 Ab in 1% BSA for 30 min. Sections were then surface stained for two hours at room temperature in PBS and washed with PBS for 30 minutes. Sections were mounted using ProLong Gold antifade reagent with DAPI (Invitrogen, Carlsbad, CA). All images were acquired with a camera (Optronics Inc., Goleta, CA) mounted on a fluorescence microscope (Olympus BX41, Leeds Precision Instruments) using a 100×/1.25 oil objective lens for a final magnification of 1000×. PictureFrame software (Optronics Inc.) was used to obtain JPEG images. The acquired digital images were processed and analyzed using Photoshop CS software (Adobe Systems).

### Adoptive Transfer

Pooled splenocytes and lymphocytes from P25 transgenic mice were CFSE labeled as previously described [84] (final CFSE concentration 1 µM; Molecular Probes). 10^6^ Vβ11+CD4+ transgenic cells were adoptively transferred intravenously via retro-orbital vein injection into recipient mice.

### Statistical Analysis

Results are given as a means SE or means SD. Comparisons between groups was done using Student *t* test analysis. Statistical significance was determined as *P* values less than .05.
